# Elevated NLRP3 Inflammasome Levels Correlate With Vitamin D in the Vitreous of Proliferative Diabetic Retinopathy

**DOI:** 10.3389/fmed.2021.736316

**Published:** 2021-10-15

**Authors:** Li Lu, Gaocheng Zou, Li Chen, Qianyi Lu, Mian Wu, Chunxia Li

**Affiliations:** ^1^Department of Ophthalmology, The First Affiliated Hospital of University of Science and Technology of China, Hefei, China; ^2^Department of Clinical laboratory, The First Affiliated Hospital of University of Science and Technology of China, Hefei, China; ^3^Department of Ophthalmology, The First Affiliated Hospital of Soochow University, Suzhou, China; ^4^Department of Endocrinology and Metabolism, The Affiliated Suzhou Hospital of Nanjing Medical University, Suzhou Municipal Hospital, Suzhou, China; ^5^Department of Ophthalmology, Shanghai TCM-Integrated Hospital, Shanghai University of TCM, Shanghai, China

**Keywords:** proliferative diabetic retinopathy (PDR), vitamin D, NLRP3 inflammasome, VEGF, vitrectomy

## Abstract

**Purpose:** This study aims to determine vitamin D concentrations in the vitreous and serum, as well as the expression levels of NLRP3 inflammasome pathway in the vitreous of patients with proliferative diabetic retinopathy (PDR). In addition, we investigated the possible correlation between NLRP3 inflammasome levels and vitamin D concentrations.

**Methods:** We obtained vitreous samples before vitrectomy from 55 PDR patients, 25 non-diabetic patients with idiopathic macular hole (IMH), and 10 non-proliferative diabetic retinopathy (NPDR) patients. We also collected serum samples from the same patients. Enzyme-linked immunosorbent assay (ELISA) was used to examine NLRP3 inflammasome pathway proteins, including NLRP3, caspase-1, IL-1β, and VEGF. In addition, vitamin D concentrations were analyzed in Roche Cobas 6000's module e601 platform using electrochemiluminescence immune assay.

**Results:** The levels of NLRP3 inflammasome pathway and VEGF increased dramatically in PDR vitreous. However, vitamin D concentrations in vitreous and serum followed the opposite trend. Meanwhile, vitreous and serum vitamin D concentrations were significantly negatively correlated with vitreous NLRP3 expression in PDR patients. Moreover, serum and vitreous vitamin D concentrations were positively correlated and demonstrated discriminatory ability in DR. The subgroup analysis of PDR group revealed that eyes with tractional retinal detachment (TRD) had higher NLRP3 inflammasome pathway and VEGF levels but lower vitamin D concentrations. Conversely, eyes that received preoperative pan-retinal photocoagulation (PRP) exhibited lower levels of NLRP3 inflammasome pathway, but vitamin D concentrations were irrelevant to laser treatment.

**Conclusions:** Our results demonstrate a strong correlation between increased NLRP3 inflammasome pathway and decreased vitamin D concentrations in the vitreous of PDR patients, which may be linked to PDR pathogenesis. In addition, vitamin D supplementation may play a key role in preventing, treating, and improving PDR prognosis due to its inhibitory impact on NLRP3 inflammasome pathway and VEGF.

## Introduction

Diabetic retinopathy (DR) is the most frequent microvascular complication of diabetes, causing blindness and vision impairment in the working-age population worldwide (1). About 35% of diabetes patients suffer from DR, 6.8% have diabetic macular edema, 7.0% have proliferative diabetic retinopathy (PDR), and 10.2% have vision-threatening DR (2). Notably, China has the highest number of diabetes mellitus (DM) patients globally, with about 116.4 million cases, implying a sizable burden of DR (3). Numerous sophisticated treatments, including anti-vascular endothelial growth factor (VEGF) therapy and vitrectomy, can minimize the vision loss of severe DR patients. However, DR pathogenesis, particularly proliferative diabetic retinopathy (PDR), remains unclear.

Much DR pathogenesis is attributed to the aberrant production of reactive oxygen species (ROS) and chronic inflammation (4, 5). Recently, our research group and Chaurasia et al. demonstrated that ROS/TXNIP/NLRP3 inflammasome pathway is a major mechanism in DR and that NLRP3 inflammasome is involved in vascular impairment in the advanced stages of the disease (6, 7). NLRP3 inflammasome is the best-characterized inflammasome, including sensor protein NLRP3, adaptor protein apoptosis-associated speck-like protein (ASC), and proinflammatory caspase, procaspase-1 (8). Upon recognition and activation by danger signals, NLRP3 inflammasome mediates caspase-1 activation, cleaving proIL-1β, and proIL-18 into their active forms (9–11). Furthermore, Loukovaara et al. discovered that caspase-1 and IL-18 levels were significantly higher in the vitreous of PDR eyes than non-proliferative diabetic retinopathy (NPDR) eyes (12), confirming the dominating role of NLRP3 in PDR pathogenesis.

In addition, our previous research found that 1,25-dihydroxy vitamin D was able to confer protection of retinal structure and function by down-regulating ROS and suppressing inflammation and apoptosis (7). The antioxidant and anti-inflammatory properties of vitamin D have been the focus of recent diabetes research (13, 14). In addition, several clinical and epidemiological studies reported an inverse relationship between retinopathy severity and serum vitamin D concentrations, with vitamin D deficiency serving as an independent determinant of DR (15–18). Therefore, based on our prior work and other studies stated above, we hypothesize that NLRP3 levels and vitamin D concentrations in vitreous fluid may be correlated and hold a critical function in DR pathogenesis.

To further investigate this issue, we conducted this study to compare NLRP3 and vitamin D levels in vitreous fluid of patients with or without PDR and analyze their relationship. Meanwhile, we evaluated the association between vitreous vitamin D concentration and serum vitamin D concentration. As a result, this study may provide advanced evidence for vitamin D as a considerable potential target for DR prevention and treatment.

## Materials and Methods

### Participants

This retrospective study was conducted following the Declaration of Helsinki and approved by the First Affiliated Hospital of University of Science and Technology of China. All participants underwent preoperative examinations, including eye examination, history taking, physiological, and laboratory blood tests. DR was diagnosed by a retinal specialist based on ICO Guidelines for Diabetic Eye Care (19). Before surgery, we excluded participants with repeated vitrectomy, vitamin D supplementation, or anti-VEGF therapy, and endocrine or digestive diseases that could affect vitamin D concentrations. In addition to main diseases of each group, we excluded patients with other ophthalmological diseases (glaucoma, serious cataract, age-related macular degeneration, rhegmatogenous retinal detachment, etc.). Finally, we excluded eyes with recent vitreous hemorrhage (<1 month) to avoid possible interference of intravitreal hemoglobin.

The study recruited 90 eyes of 90 inpatients from the ophthalmology department of the First Affiliated Hospital of University of Science and Technology of China, between June 2019 and September 2019. After that, the patients were divided into three groups: the control group (25 eyes of 25 patients with idiopathic macular hole), NPDR group (10 eyes of 10 patients with NPDR), and PDR group (55 eyes of 55 patients with PDR). None of these control patients had any severe systemic medical problems, including diabetes, while all patients in NPDR and PDR groups had type 2 diabetes (T2DM). The demographic details of patients are summarized in Table 1.

**Table 1:** Systemic and ocular characteristics of participants.

	**Control (*n* = 25)**	**NPDR (*n* = 10)**	**PDR (*n* = 55)**	***P*** **-value**		
				***P* ^a^**	***P* ^b^**	***P* ^c^**
**Patient characteristics**						
Age (years)	61.68 ± 10.63	65.50 ± 9.30	51.07 ± 11.50	>0.99	<0.01	<0.01
Female/male (*n*)	16/9	5/5	28/27	0.9519	0.9519	0.1365
Duration of diabetes (years)	–	12.6 ± 5.89	13.0 ± 6.46	–	–	0.856
HbA1c (%)	5.27 ± 0.35	5.97 ± 0.48	7.82 ± 1.55	0.1801	<0.0001	0.0041
Body mass index (kg/m2)	23.67 ± 3.28	22.98 ± 2.41	23.74 ± 2.68	>0.99	>0.99	>0.99
TC (mmol/L)	4.52 ± 0.71	5.49 ± 0.59	4.99 ± 1.14	0.1272	0.0113	0.2515
TG (mmol/L)	1.76 ± 0.71	2.12 ± 2.23	1.71 ± 1.14	>0.99	0.9274	>0.99
HDL (mmol/L)	1.13 ± 0.20	1.025 ± 0.24	1.05 ± 0.28	0.6994	0.4579	>0.99
LDL (mmol/L)	2.25 ± 0.66	2.72 ± 0.70	2.47 ± 0.73	0.2083	0.7058	0.7588
Systolic BP (mmHg)	136.10 ± 20.49	147.30 ± 12.89	140.90 ± 19.60	0.1062	0.8543	0.3714
Diastolic BP (mmHg)	82.08 ± 8.36	80.20 ± 7.42	83.20 ± 13.10	>0.99	>0.99	>0.99
**Ocular characteristics**						
Vitreous hemorrhage	–	0/10	55/55	–	–	<0.01
Tractional retinal detachment	–	0/10	21/55	–	–	<0.01
Previous laser treatment	–	6/10	28/55	–	–	0.7359
(Non-PRP/IRP/PRP)	–	4/3/3	27/9/19	–	–	
Epiretinal fibrosis	–	–	41/55	–	–	–
silicon oil filling	–	–	21/55	–	–	–
Operated eye (right/left)	8/17	5/5	20/35	0.9519	0.9519	0.1365
NLRP3 (ng/mL)	0.03 ± 0.00	0.04 ± 0.00	0.05 ± 0.01	0.0615	<0.0001	0.0174
Caspase-1 (pg/mL)	15.48 ± 3.8	12.38 ± 2.97	44.52 ± 12.32	>0.99	<0.0001	<0.0001
IL-1β (pg/mL)	8.72 ± 5.22	11.07 ± 3.56	29.03 ± 6.17	>0.99	<0.0001	<0.0001
VEGF (pg/mL)	30.98 ± 5.88	56.31 ± 11.36	350.54 ± 81.03	0.2610	<0.0001	0.0007
SVD (ng/mL)	19.83 ± 4.51	17.74 ± 4.47	14.46 ± 5.73	>0.99	<0.0001	0.1603
VVD (ng/mL)	21.83 ± 3.95	16.86 ± 2.40	18.21 ± 2.91	0.0049	0.0008	>0.99

a *P, Control vs. NPDR*;

b *P, Control vs. PDR*;

c *P, NPDR vs. PDR*.

*Data are displayed either as mean ± SD or numbers of subjects. P ≤ 0.05 is considered statistically significant. PDR, proliferative diabetic retinopathy; TC, total cholesterol; TG, triglyceride; HDL, high-density lipoprotein; LDL, low-density lipoprotein; PRP, pan-retinal photocoagulation; IRP, incomplete retinal laser photocoagulation; IL-1β, interleukin-1β; VEGF, vascular endothelial growth factor; SVD, serum 25 (OH) D; VVD, vitreous 25 (OH) D*.

### Vitrectomy and Biological Samples Collection

The patient's blood and vitreous samples were collected during the same period of hospitalization. All patients among the three groups underwent a 23-gauge standard three-port pars plana vitrectomy without an infusion of artificial fluid using CONSTELLATION Vision System (Alcon Laboratories, Inc., Fort Worth, TX, USA). The vitreous fluid (0.3–0.5 mL) was collected by manual aspiration into a syringe via the vitrectomy with the cutting function activated (12). Vitreous samples were immediately centrifuged at 13,000 rpm for 5 min at 4°C in sterile 1.5 mL Eppendorf tubes. Supernatants were rapidly collected and stored at −80°C until the assay was performed (20). After fasting overnight for at least 10 h, blood samples of patients were taken by aseptic vein puncture and submitted for testing.

### Laboratory Assessments

The blood samples of subjects underwent the following tests: HbA1c, triglyceride (TG), total cholesterol (TC), high-density lipoprotein cholesterol (HDL-C), and low-density lipoprotein cholesterol (LDL-C) using an automatic biochemical analyzer (FUJI DRI-CHEM 4000i, Fuji, Japan). In addition, all participants were evaluated clinically and in the laboratory according to established procedures.

### Measurement of NLRP3 Inflammasome Pathway and VEGF by Enzyme-Linked Immunosorbent Assay (ELISA)

The vitreous NLRP3 levels were measured using a commercially available ELISA kit (Cusabio, Wuhan, China), following the manufacturer's instructions. Moreover, IL-1β, caspase-1, and VEGF levels in the vitreous fluid were also determined using ELISA following the manufacturer's instructions (R&D Systems, Minneapolis, MN, USA).

### 25 (OH) D Measurements

Vitreous and serum 25 (OH) D concentrations were determined using Roche Cobas electrochemiluminescence immunoassay. The samples were processed in a single batch in duplicate on each analyzer according to manufacturer's instructions. A total of 200 μL of each vitreous or serum sample was used per assay without dilution. The calibration curves were constructed using calibrators provided in the kits. Roche Cobas Vitamin D total assay had a measurement range of 2.9–63.4 ng/mL (21).

### Statistical Analysis

GraphPad Prism 7.0 Software (GraphPad, San Diego, CA, USA) was deployed to accomplish all data analysis and graph drawing. The measurement data were presented as mean ± SD. The Mann–Whitney *U*-test was employed to compare the parameters of the two groups. The Kruskal–Wallis test followed by Dunns was employed to compare the three groups among each other. Spearman correlation coefficients were used to determine correlations. A receiver-operating characteristic (ROC) curve was used to estimate the predictive value of the vitreous or serum 25 (OH) D concentrations. For all comparisons, a value of *P* < 0.05 was considered statistically significant.

## Results

### Patients Characteristics

In this study, the patients were randomly assigned to control, NPDR, and PDR groups. Table 1 summarizes the basic systemic and ocular characteristics. Among the three groups, no significant difference was observed in gender, body mass index (BMI), or blood pressure. While the mean age of patients in PDR group was 51.07 ± 11.50, this was significantly lower than that in control (61.68 ± 10.63, *P* < 0.01) and NPDR (65.50 ± 9.30, *P* < 0.01) groups. In addition, the PDR group had a higher HbA1c level than NPDR group. Regarding the ocular characteristics, vitreous hemorrhage, tractional retinal detachment (TRD), previous laser treatment, epiretinal fibrosis, and silicon oil filling were recorded in NPDR and PDR groups after operations.

### Quantitative Analysis of NLRP3 Inflammasome Pathway, 25 (OH) D Concentration, and VEGF

**Figure 1:**
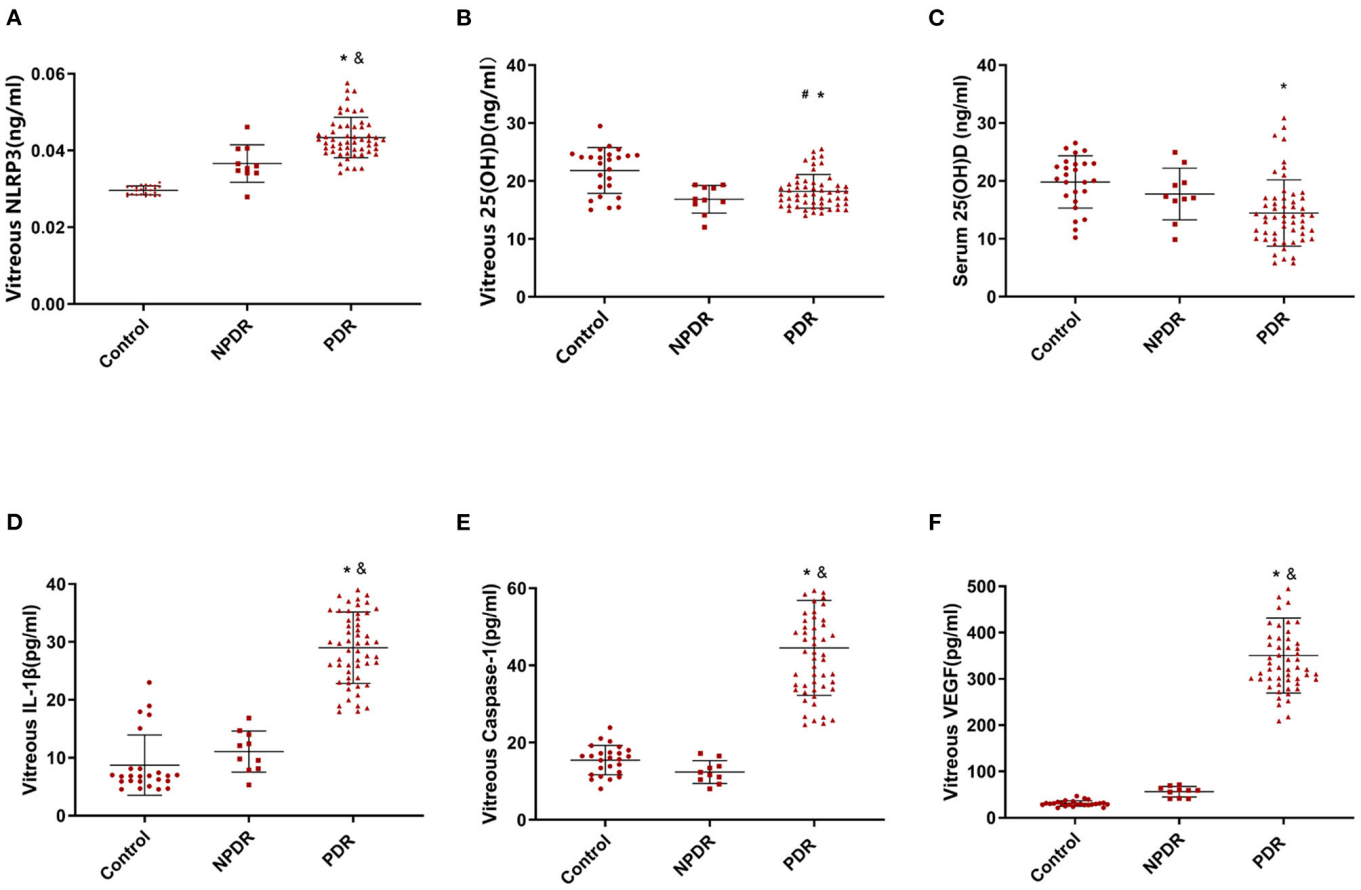
The levels of NLRP3 and the concentrations of serum and vitreous 25 (OH) D in three groups. **(A)** Vitreous NLRP3 levels in PDR, NPDR, and control groups. **(B)** Vitreous 25 (OH) D concentrations in PDR, NPDR, and control groups. **(C)** Serum 25 (OH) D concentrations in PDR, NPDR, and control groups. **(D)** Vitreous IL-1β levels in PDR, NPDR, and control groups. **(E)** Vitreous caspase-1 levels in PDR, NPDR, and control groups. **(F)** Vitreous VEGF levels in PDR, NPDR, and control groups. ^#^*P* < 0.01, Control vs. NPDR; ^*^P < 0.01, Control vs. PDR; ^&^*P* < 0.01, NPDR vs. PDR.

We evaluated the expression of NLRP3 inflammasome pathway, including NLRP3, caspase-1, and IL-1β in vitreous, as well as the concentration of 25 (OH) D both in vitreous and serum among the three groups (Table 1). We found that PDR group had significantly higher NLRP3 expression than the control group. Moreover, PDR eyes had a much higher expression of NLRP3 than NPDR eyes (Figure 1A). Meanwhile, 25 (OH) D concentrations were significantly lower in the PDR group than in the control group, both in serum and vitreous fluid (Figures 1B,C). Finally, the expression of IL-1β, caspase-1, and VEGF followed the same pattern as NLRP3 (Figures 1D–F).

### Correlation Analysis of Vitreous 25 (OH) D Concentrations and Serum 25 (OH) D Concentrations as Well as Their Predictive Value for DR

**Figure 2:**
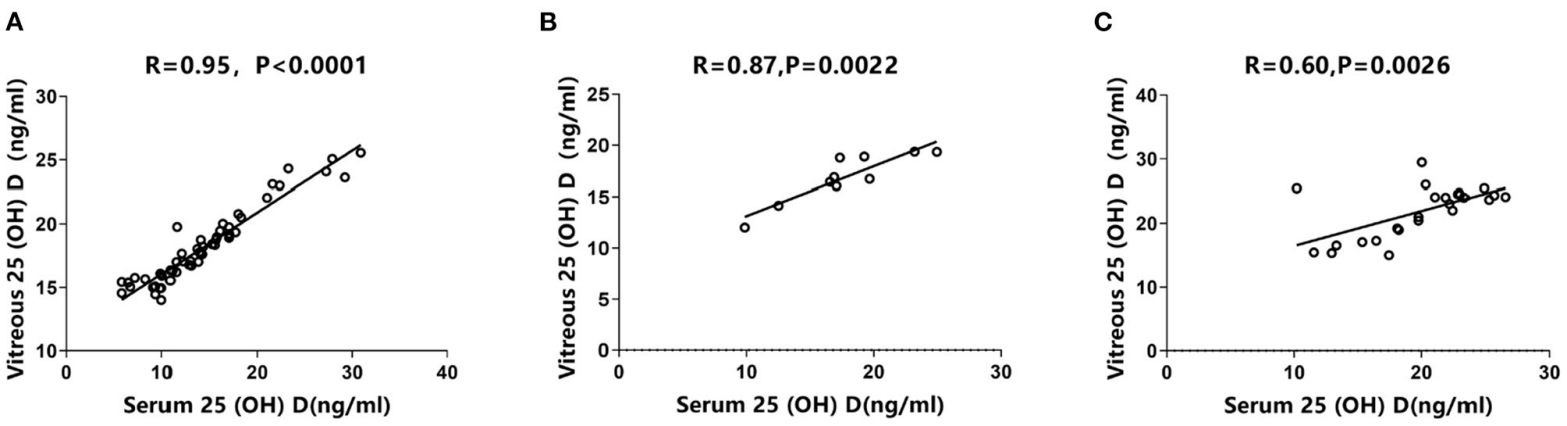
Correlations between vitreous and serum 25 (OH) D concentrations. Spearman's correlation tests showed positively significant correlations **(A)** in PDR group (*R* = 0.95, *P* < 0.0001); **(B)** in NPDR group (*R* = 0.87, *P* = 0.0022), and **(C)** in control group (*R* = 0.60, *P* = 0.0026).

**Figure 3:**
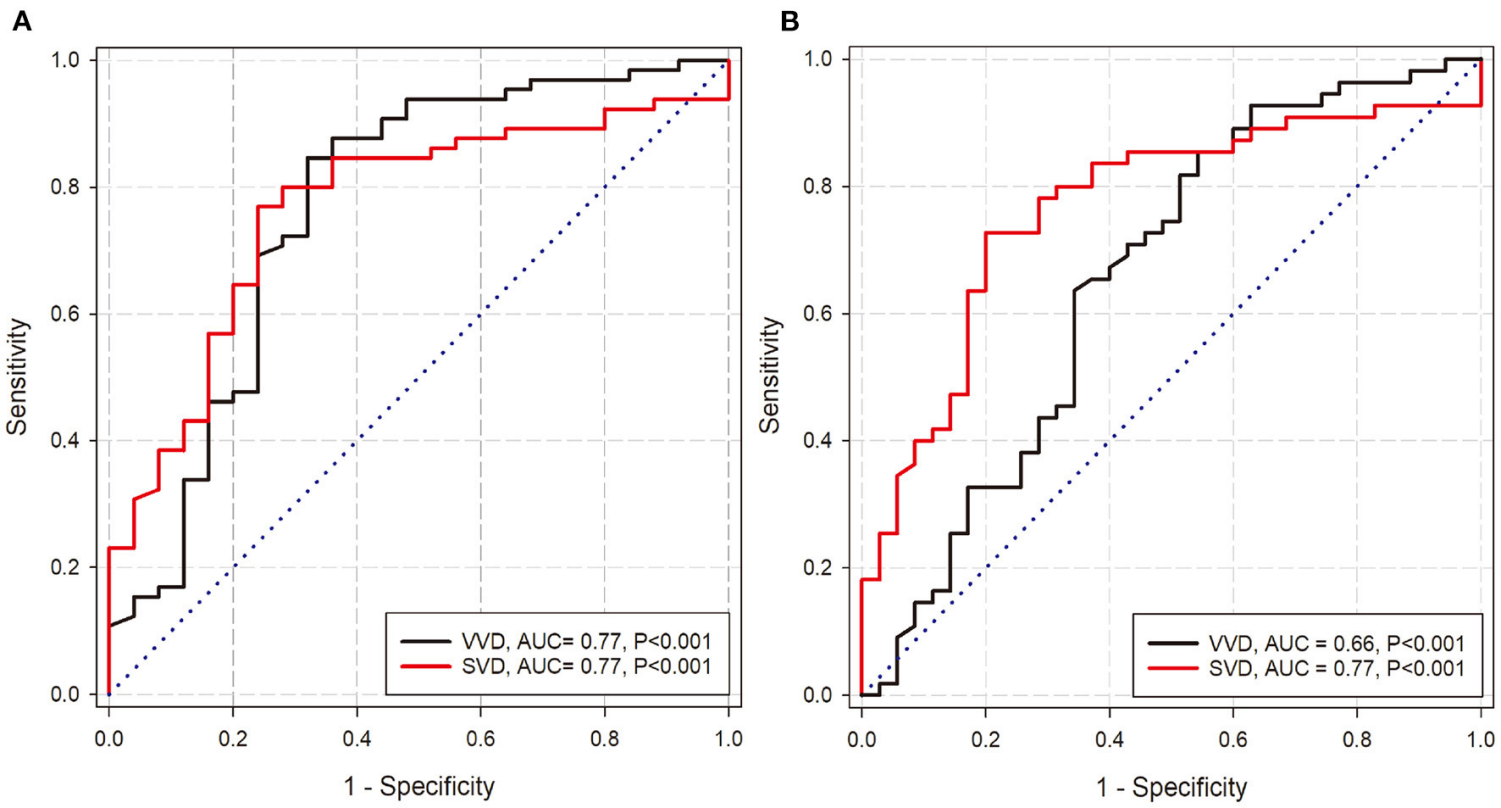
Prediction of DR and PDR by vitreous and serum 25 (OH) D. **(A)** The ROC-curve of the serum 25 (OH) D and vitreous 25 (OH) D for predicting DR. **(B)** The ROC-curve of the serum 25 (OH) D and vitreous 25 (OH) D for predicting PDR. [SVD: serum 25 (OH) D; VVD: vitreous 25 (OH) D].

Using Spearman's correlation test, the three groups exhibited a significant positive correlation between vitreous and serum 25 (OH) D concentrations. Specifically, the PDR group had the highest correlation (*R* = 0.95, *P* < 0.0001, Figure 2A), which was slightly higher than NPDR group (*R* = 0.87, *P* = 0.0022, Figure 2B). Conversely, the control group exhibited a relatively weak positive correlation between vitreous and serum 25 (OH) D concentrations (*R* = 0.60, *P* = 0.0026, Figure 2C). According to ROC-curve analyses, both serum and vitreous 25 (OH) D showed discriminatory ability in predicting DR (NPDR and PDR) and PDR. In DR prediction, they obtained the same area under curve (AUC) of 0.77 (Figure 3A). In particular, serum 25 (OH) D has a better predictive value (AUC: 0.77) than serum 25 (OH) D (AUC: 0.66) in PDR prediction (Figure 3B).

### Correlation Analysis of Vitreous NLRP3 Pathway Levels and 25 (OH) D Concentrations

**Figure 4:**
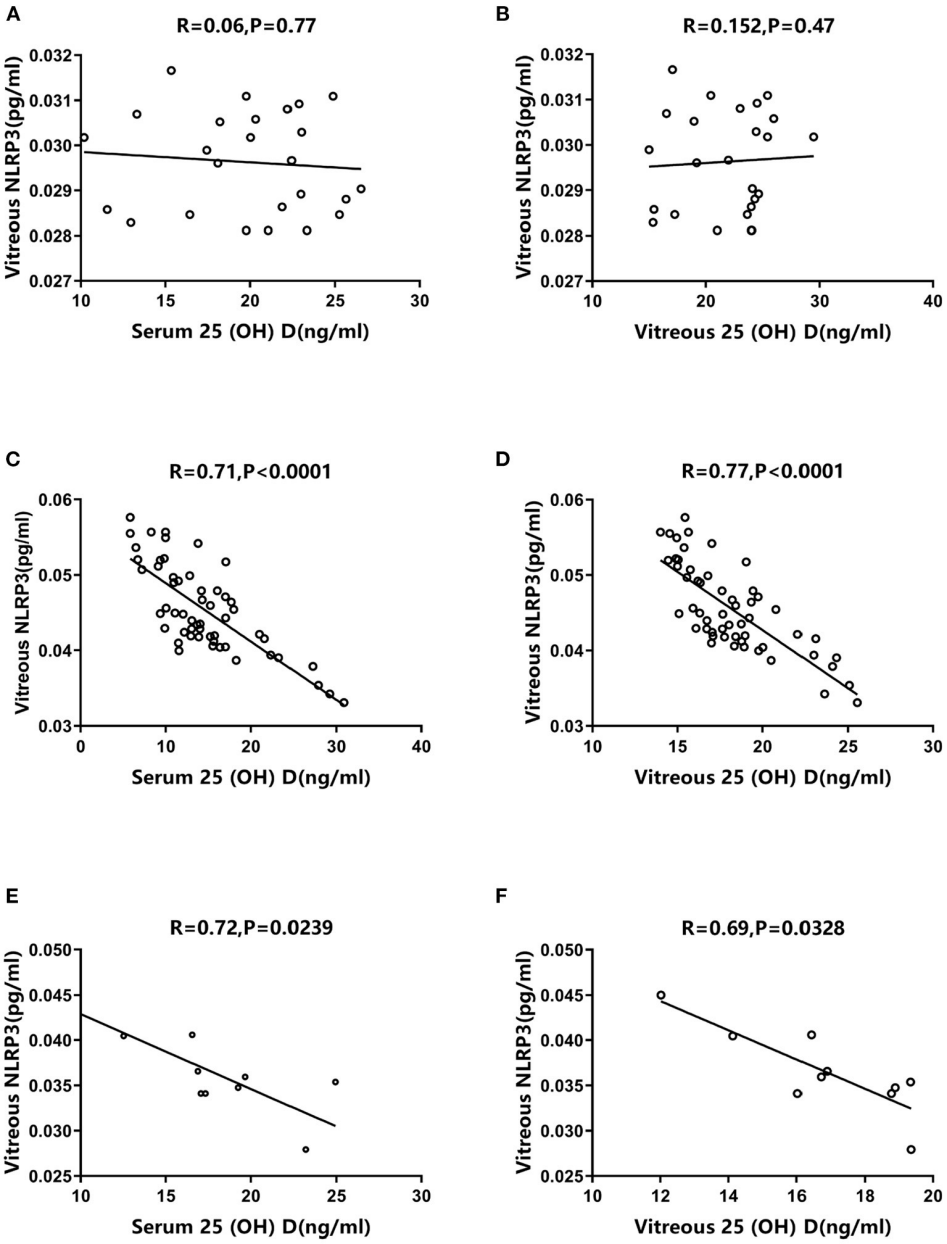
Correlations between vitreous NLRP3 levels and serum/vitreous 25 (OH) D concentrations. Spearman's correlation tests exhibited no significant correlations **(A)** between vitreous NLRP3 level and serum 25 (OH) D concentration in control group (*R* = 0.06, *P* = 0.77), and **(B)** between vitreous NLRP3 level and vitreous 25 (OH) D concentration in control group (*R* = 0.152, *P* = 0.47). Spearman's correlation tests presented negatively significant correlations **(C)** between vitreous NLRP3 level and serum 25 (OH) D concentration in PDR group (*R* = 0.71, *P* < 0.0001), **(D)** between vitreous NLRP3 level and vitreous 25 (OH) D concentration in PDR group (*R* = 0.77, *P* < 0.0001), **(E)** between vitreous NLRP3 level and serum 25 (OH) D concentration in NPDR group (*R* = 0.72, *P* = 0.0239) and **(F)** between vitreous NLRP3 level and vitreous 25 (OH) D concentration in NPDR group (*R* = 0.69, *P* = 0.0328).

**Figure 5:**
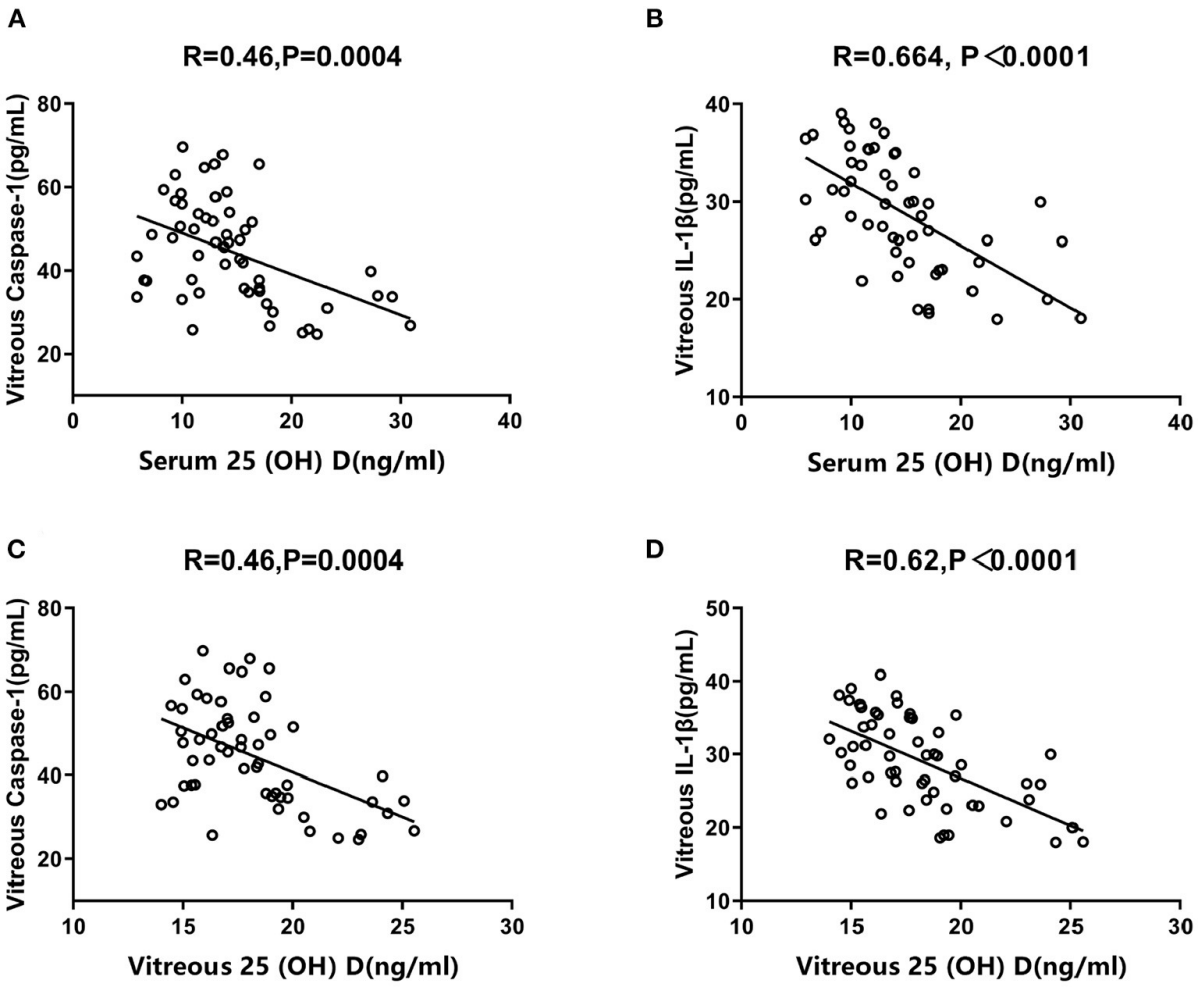
Correlations between vitreous Caspase-1/IL-1β levels and serum/vitreous 25 (OH) D concentrations in PDR group. Spearman's correlation tests presented negatively significant correlations **(A)** between vitreous Caspase-1 level and serum 25 (OH) D concentration (*R* = 0.46, *P* = 0.0004), **(B)** between vitreous IL-1β level and serum 25 (OH) D concentration (*R* = 0.664, *P* < 0.0001), **(C)** between vitreous Caspase-1 level and vitreous 25 (OH) D concentration (*R* = 0.46, *P* = 0.0004) and **(D)** between vitreous IL-1β level and vitreous 25 (OH) D concentration (*R* = 0.62, *P* < 0.0001).

The correlations between vitreous NLRP3 levels and 25 (OH) D concentrations varied significantly among the three groups. The control group exhibited no significant correlation between vitreous NLRP3 levels and serum/vitreous 25 (OH) D concentrations (*R* = 0.06, *P* = 0.77; *R* = 0.152, *P* = 0.47, Figures 4A,B). However, the PDR group exhibited a negative association between vitreous NLRP3 levels and both serum and vitreous 25 (OH) D concentrations (*R* = 0.71, *P* < 0.0001; *R* = 0.77, *P* < 0.0001, Figures 4C,D). The same negative correlations were also observed in NPDR group (*R* = 0.72, *P* = 0.0239; *R* = 0.69, *P* = 0.0328, Figures 4E,F). Moreover, in PDR group, we tested the correlation between 25 (OH) D concentrations and the downstream effects of NLRP3 such as IL-1β and caspase-1 levels. Again, the negative correlation trends were observed between the downstream of NLRP3 pathway and 25 (OH) D concentrations (Figure 5).

### Subgroup Analysis of PDR Group

Based on various ocular characteristics observed during operations, we further performed subgroup analyses from two perspectives in PDR group. First, based on TRD presence, we divided the results into two parts and compared the levels of NLRP3 inflammasome pathway, 25 (OH) D, and VEGF in vitreous fluid (Table 2). We found that TRD eyes (*n* = 21) had significantly higher levels of NLRP3 (*P* < 0.0001), caspase-1 (*P* = 0.0182), IL-1β (*P* = 0.0007), and VEGF (*P* = 0.0011) and lower concentrations of 25 (OH) D (*P* < 0.0001). Second, we classified patients into three groups depending on their past laser treatment status, including no laser treatment group, incomplete pan-retinal photocoagulation group, and pan-retinal photocoagulation (PRP) group. PRP is defined following our previously published study (22). Then, the factors compared in the first perspective were compared in the second perspective. All above proteins were considerably decreased in the PRP group compared with the no laser treatment group, while 25 (OH) D concentrations did not differ significantly across the three groups (Table 3).

**Table 2:** Level of inflammatory factor and vitreous 25 (OH) D in PDR patients with or without tractional retinal detachment.

	**TRD (*n* = 21)**	**Non-TRD (*n* = 34)**	***P*-value**
NLRP3 (ng/mL)	0.05 ± 0.01	0.04 ± 0.00	<0.0001
VVD (ng/mL)	16.14 ± 1.44	19.49 ± 2.85	<0.0001
Caspase-1 (pg/mL)	49.26 ± 9.45	41.60 ± 13.09	0.0182
IL-1β(pg/mL)	32.48 ± 4.33	26.89 ± 6.21	0.0007
VEGF (pg/mL)	387.60 ± 71.55	327.60 ± 78.90	0.0011

*Data are presented as mean ± SD. P <0.05 is considered statistically significant*.

*TDR, tractional retinal detachment; VVD, vitreous 25 (OH) D; IL-1β, interleukin-1β; VEGF, vascular endothelial growth factor*.

**Table 3:** Level of inflammatory factor and vitreous 25 (OH) D in PDR patients according to pan-retinal laser photocoagulation state.

	**Non-PRP (*n* = 27)**	**IRP (*n* = 9)**	**PRP (*n* = 19)**	***P*** **-value**		
				***P* ^a^**	***P* ^b^**	***P* ^c^**
NLRP3 (ng/mL)	0.05 ± 0.01	0.04 ± 0.01	0.04 ± 0.00	0.0752	0.0017	>0.99
VVD (ng/mL)	17.67 ± 2.88	18.62 ± 3.76	18.78 ± 2.50	>0.99	0.3051	>0.99
Caspase-1 (pg/mL)	49.68 ± 10.71	45.77 ± 9.46	36.60 ± 11.96	>0.99	0.0006	0.1141
IL-1β (pg/mL)	31.56 ± 5.65	30.56 ± 5.37	24.70 ± 4.96	>0.99	0.0003	0.0673
VEGF (pg/mL)	394.40 ± 80.45	341.80 ± 41.37	292.30 ± 55.52	0.4580	<0.0001	0.0971

a *P, Non-PRP vs. IRP*;

b *P, Non-PRP vs. PRP*;

c *P, IRP vs. PRP*.

*Data are shown as mean ± SD. P < 0.05 was considered statistically significant*.

*PRP, pan retinal photocoagulation; IRP, incomplete retinal laser photocoagulation; VVD, vitreous 25 (OH) D; IL-1β, interleukin-1β; VEGF, vascular endothelial growth factor*.

## Discussion

Vitamin D, a steroid hormone, is critical for calcium and bone homeostasis. Extraskeletal effects have also been documented (23), including regulating cardiovascular homeostasis, tuning immunity systems, and modulating inflammation (24, 25). Furthermore, several clinical research studies have indicated the link between DR characterized by persistent low-grade inflammation and vitamin D deficiency (15, 26). This study confirmed that PDR patients had significantly lower vitreous or serum 25 (OH) D concentrations and higher vitreous levels of NLRP3 inflammasome than those without DM. Meanwhile, we observed a significant positive correlation between vitreous and serum 25 (OH) D concentrations in the three groups and a negative correlation between vitreous NLRP3 levels and serum/vitreous vitamin D concentrations in NPDR and PDR groups. To the best of our knowledge, this is the first report to evaluate vitamin D concentrations in human vitreous fluid. Our current findings imply that vitamin D deficiency may be a possible mechanism to activate NLRP3 inflammasome pathway during PDR pathogenesis.

The tendency of vitamin D observed in the present study was consistent with other studies on DM or diabetic complications (27–29). The majority of those studies have concluded that the link between vitamin D and DM as well as its complications is mainly because vitamin D deficiency contributes to their key pathological processes through multiple mechanisms such as enhancement of insulin resistance and β-cell death (30). In other words, vitamin D deficiency is a factor in DM development and its complications. Vitamin D deficiency and insufficiency is a global health problem. About 30% of children and 60% of adults worldwide are vitamin D deficient and insufficient, respectively (31). In this study, even the serum vitamin D concentrations of the control patients (19.83 ± 4.51) were defined as vitamin D deficiency (32). The main cause of vitamin D deficiency is inadequate dietary intake and lack of exposure to sunlight, which is the main source of vitamin D synthesis for most children and adults (33). As PDR patients with the highest BMI of the three groups, T2DM-related obesity may lead to vitamin D dilution in body fat and exacerbate vitamin D deficiency (34). Besides, vitamin D deficiency is also an issue affecting people of all ages, especially the elderly. Individuals older than 51 years have less sun exposure and a weaker capacity of the skin to produce vitamin D (32). However, in this study, most participants were older than 51 years, indicating that age was not the primary factor for the difference in vitamin D concentrations between PDR and control groups. Most PDR patients hospitalized for surgery were in the working population, whereas IHM patients in the control group tended to be older when they underwent surgery. It is well-known that the vitreous becomes more liquefied with age (35). During surgery, we found that vitreous liquefaction occurred in most participants, resulting in a more uniform distribution of factors tested in the vitreous fluid.

Other studies have also focused on vitamin D's impact on inflammation, which has been demonstrated to reduce the numbers of activated macrophages and chronic inflammatory response in retina (36). On the other hand, vitamin D deficiency might cause increased production of proinflammatory cytokines in PDR patients (37). However, combining our present and previous studies has enabled us to thoroughly discuss the underlying mechanisms of vitamin D's role in DR. *In vivo*, we demonstrated that vitamin D3 could maintain normal retinal structure and retinal vascular function and suppress apoptosis of retinal cells in diabetic rats. While, *in vitro*, we provided evidence that vitamin D3 decreased ROS and further suppressed ROS/TXNIP/NLRP3 inflammasome pathway in high-glucose-induced retinal microvascular endothelial cells (7). As a result, our prior findings partially explained the differences in NLRP3 levels and 25 (OH) D concentrations among the three groups in the current study, while the correlation between vitreous NLRP3 levels and serum/vitreous 25 (OH) D concentrations also provides further evidence for previous animal and cellular research.

Despite the above-mentioned principal conclusions, several valuable analyses were conducted and yielded intriguing findings. First, we performed additional correlation analysis of vitreous and serum 25 (OH) D concentrations in the three groups and obtained positive correlation results in each case. Interestingly, the correlation degree appears to be linked to DR severity, with the highest correlation observed in PDR group (*R* = 0.95). There is a dearth of evidence reporting the correlation between vitamin D levels in different body fluids. Lin et al. reported that following 8 weeks of vitamin D supplementation, the elevated vitamin D levels in the tear fluid and aqueous humor were parallel with increased plasma concentrations in rabbits, but this was not found in the vitreous fluid (38). We believe that the dominant reason for this discrepancy is that blood-retinal barrier (BRB) was destroyed in PDR patients, facilitating vitamin D shift due to higher vascular permeability (39). Simultaneously, ROC-curve analyses revealed that serum 25(OH) D performed well in predicting DR, especially PDR. Thus, serum 25(OH) D concentration could be an accessible potential diagnostic marker of PDR. Second, a subgroup analysis was performed in PDR group based on two dimensions: whether the patient presented with TRD and whether the patient received preoperative laser photocoagulation treatment. We found that PDR eyes with TRD had significantly lower concentrations of vitreous 25 (OH) D and higher levels of NLRP3 inflammasome pathway as well as VEGF (Table 2). Angiogenesis and fibrous tissue proliferation are critical in diabetic TRD pathophysiology (40, 41). Undoubtedly, VEGF is the most crucial factor in this process. In addition, activating NLRP3 inflammasome pathway is required for fibrosis development (42). Notably, vitamin D_3_ can concurrently suppress NLRP3 inflammasome pathway while decreasing VEGF level, as demonstrated by our results from diabetic animals (7), which could also account for our present findings. Preoperative PRP may be considered as an effective DR treatment, particularly if the eye never received any laser or if the previous laser appears inadequate. As a result, it prevents TRD by inhibiting neovascularization and stabilizes PDR eyes with TRD. The main mechanism underlying this effect is to produce oxygen benefit in the treated area of retina, thereby alleviating the elevated VEGF levels in diabetic retina (22, 43). Whereas, vitreous 25 (OH) D concentrations did not appear to be influenced by PRP's completion, with no significant difference among the three groups (Table 3). We speculate that instantaneous thermal effect generated by laser targeting neuroretina and retinal pigment epithelium (RPE) will have little influence on the vitreous during laser irradiation (44). There is a possibility that vitamin D supplementation could play a greater role in conjunction with PRP treatment.

Several limitations remain in the current study. To better reflect the actual pathological changes in the retinal microenvironment, we evaluated 25 (OH) D concentrations and NLRP3 inflammasome pathway levels, mainly in the vitreous. Although we excluded eyes with recent vitreous hemorrhage (<1 month), the slower degrading blood components may still pose a potential contamination risk to the vitreous sample. Unlike our earlier research, which employed the activated metabolite, 1,25-dihydroxy vitamin D_3_ as a target, the present study utilized the best marker of vitamin D status, 25 (OH) D, to measure vitamin D concentrations in human tissues (45), benefiting from its longer half-life and better stability (46).

In conclusion, our current study confirmed NLRP3 inflammasome pathway activation and serum/vitreous vitamin D deficiency in PDR patients. In particular, we found a significant positive correlation between vitamin D and the inflammatory factor NLRP3. By combining our prior findings in diabetic rats and high glucose-induced human retinal microvascular endothelial cells, we partly demonstrated that decreased NLRP3 inflammasome pathway levels caused by vitamin D have protective effects on DR, particularly PDR pathogenesis. Additionally, the link between vitreous and serum vitamin D concentrations and ROC-curve analyses may offer a potentially accessible diagnostic marker of PDR. Moreover, subgroup analyses highlighted the clinical value of vitamin D during PDR treatment. All above results demonstrate the critical importance of vitamin D supplementation in preventing DR onset and/or progression.

## Data Availability Statement

The raw data supporting the conclusions of this article will be made available by the authors, without undue reservation.

## Ethics Statement

The studies involving human participants were reviewed and approved by the Ethics Committee of the First Affiliated Hospital of University of Science and Technology of China. Written informed consent for participation was not required for this study in accordance with the national legislation and the institutional requirements.

## Author Contributions

LL and CL: design of the study. GZ, LC, QL, and MW: acquisition, analysis, and interpretation of data. LL and GZ: drafting of the manuscript. LL and MW: statistical analysis. LL, QL, and MW: obtaining the fund. CL: supervising the process. All authors revision and approval of the manuscript.

## Funding

This study was supported by grants from the Fundamental Research Funds for the Central Universities (grant no. WK9110000099), the Natural Science Foundation of Jiangsu Province (grant no. BK20200211), and the Young Talent Program of Gusu Health Project (grant no. GSWS2020014).

## Conflict of Interest

The authors declare that the research was conducted in the absence of any commercial or financial relationships that could be construed as a potential conflict of interest.

## Publisher's Note

All claims expressed in this article are solely those of the authors and do not necessarily represent those of their affiliated organizations, or those of the publisher, the editors and the reviewers. Any product that may be evaluated in this article, or claim that may be made by its manufacturer, is not guaranteed or endorsed by the publisher.
